# Extensive cerebral and extracerebral metastases from a large-cell neuroendocrine cervical carcinoma

**DOI:** 10.11604/pamj.2017.28.264.14347

**Published:** 2017-11-24

**Authors:** Mauricio Fernando Villamar

**Affiliations:** 1 Department of Neurology, University of Kentucky, Lexington, KY, USA

**Keywords:** Cervical cancer, metastasis, brain tumors, neuroendocrine, oncology

## Image in medicine

**Figure 1:**
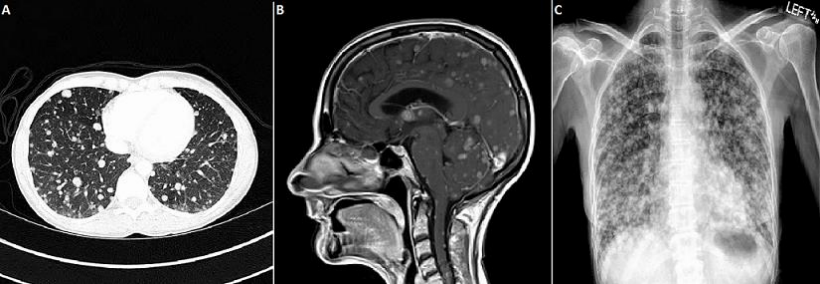
Extensive metastases from large-cell neuroendocrine carcinoma of the cervix: A) chest CT with iodinated contrast; B) sagittal post-gadolinium T1-weighted MRI of the brain and cervical cord; C) chest X-ray

A 43-year-old woman, gravida 2 para 2 with no regular medical care, presented for 3 months of pelvic pain and vaginal bleeding. Pelvic examination revealed an 8-cm necrotic cervical mass. Biopsy of the lesion demonstrated high-grade large-cell neuroendocrine carcinoma (LCNEC) of the cervix. CT with contrast of chest, abdomen and pelvis showed extensive pulmonary (Figure 1A), hepatic and renal metastases (Stage IVB). She received palliative radiation, 6 cycles of etoposide and cisplatin, and 1 cycle of bevacizumab. Seven months after diagnosis she developed intermittent headaches and expressive aphasia. MRI, pre-gadolinium and post-gadolinium, revealed numerous parenchymal and leptomeningeal contrast-enhancing lesions affecting brain and spinal cord (Figure 1B). Chest X-ray re-demonstrated innumerable lung metastases (Figure 1C). The patient decided to pursue hospice care and died 1 month later. Brain metastases from cervical cancer are exceedingly rare. However, brain metastases can occasionally occur from neuroendocrine cervical carcinomas, which account for up to 2% of all cervical cancers. LCNEC is an aggressive, poorly differentiated neoplasm with high mitotic rate, lymphovascular space involvement, and extensive necrosis. It metastasizes early. Due to the rarity of LCNEC, there are no randomized controlled trials evaluating therapies. Clinical guidelines suggest that treatment with surgical resection followed by platinum-and etoposide-based combination chemotherapy can improve survival in early stages. Still, LCNEC has poor prognosis. In a series of 62 patients, median overall survival for stage I, II, III, and IV LCNEC was 19, 17, 3 and 1.5 months, respectively.

